# Iron Supplementation Interferes With Immune Therapy of Murine Mammary Carcinoma by Inhibiting Anti-Tumor T Cell Function

**DOI:** 10.3389/fonc.2020.584477

**Published:** 2020-12-04

**Authors:** Piotr Tymoszuk, Manfred Nairz, Natascha Brigo, Verena Petzer, Simon Heeke, Brigitte Kircher, Natascha Hermann-Kleiter, Victoria Klepsch, Igor Theurl, Günter Weiss, Christa Pfeifhofer-Obermair

**Affiliations:** ^1^ Department of Internal Medicine II, Medical University of Innsbruck, Innsbruck, Austria; ^2^ Department of Internal Medicine V, Medical University of Innsbruck, Innsbruck, Austria; ^3^ Institute for Research on Cancer and Aging, Laboratory of Clinical and Experimental Pathology (LPCE), Hôpital Pasteur, Nice, France; ^4^ Division of Translational Cell Genetics, Medical University of Innsbruck, Innsbruck, Austria; ^5^ Christian Doppler Laboratory for Iron Metabolism and Anemia Research, Medical University of Innsbruck, Innsbruck, Austria

**Keywords:** T cell, immunotherapy, cancer prognosis, iron, immune checkpoint, mammary carcinoma

## Abstract

Iron is both, an essential compound for many metabolic processes, and iron deficiency can impact on the proliferation of cells including lymphocytes but also tumor cells. On the other hand, excess iron-catalyzed radical formation can induce cellular toxicity which has been previously demonstrated for T cells in hereditary iron overload. Despite these interconnections, little is known on the effects of clinically approved intravenous iron supplements for curing cancer-related anemia, on T cell differentiation, tumor proliferation, anti-tumor T cell responses and, of clinical importance, on efficacy of cancer immunotherapies. Herein, we analyzed the effects of intravenous iron supplementation on T cell function and on the effectiveness of anti-cancer chemotherapy with IL-2/doxorubicin or immunotherapy with checkpoint-inhibitor anti-PD-L1 in C57Bl/6N female mice with implanted E0771 mammary carcinomas. We found that iron application resulted to an increased availability of iron in the tumor microenvironment and stimulation of tumor growth. In parallel, iron application inhibited the activation, expansion and survival of cytotoxic CD8^+^ T cells and of CD4^+^ T helper cells type 1 and significantly reduced the efficacy of the investigated anti-cancer treatments. Our results indicate that iron administration has a tumor growth promoting effect and impairs anti-cancer responses of tumor infiltrating T lymphocytes along with a reduced efficacy of anti-cancer therapies. Iron supplementation in cancer patients, especially in those treated with immunotherapies in a curative setting, may be thus used cautiously and prospective studies have to clarify the impact of such intervention on the outcome of patients.

## Introduction

Because of its high redox activity iron is a key component of several enzymatic processes. Virtually every cell of the body, including malignant cells, requires iron for its metabolism and proliferation. Especially, the production of hemoglobin during erythropoiesis consumes about 20–30 mg of iron per day and additional iron is needed for the synthesis of several enzymes. Most iron is provided by macrophages which ingest aged or damaged red blood cells (1). After phagocytosis, the heme of erythrocyte hemoglobin is mobilized to the cytoplasm, degraded by heme oxygenase 1, and molecular iron is exported from the macrophage *via* the iron-exporter ferroportin-1 to the circulation, a process which is negatively controlled by the hormone hepcidin (2). Iron in the circulation is transported bound to transferrin and is taken up by metabolically active and dividing cells *via* transferrin receptor-1 (3). The uptake of iron *via* transferrin receptor-1 is thus of highest relevance for the differentiation of rapidly dividing cells such as erythroblasts and lymphocytes (4, 5). As a consequence, mutations in the gene coding for transferrin receptor-1, *TFRC*, can cause combined immunodeficiency characterized by impaired function of B and T lymphocytes (6). On the other hand, an excess of intracellular iron in cells has to be stored within ferritin to avoid toxicity of labile iron *via* catalysis of hydroxyl radical formation (7, 8). Since iron is crucial for both microbes and mammalian cells, iron homeostasis undergoes subtle changes during infection and inflammatory processes resulting in sequestration of the metal within macrophages, thereby reducing circulating iron pools and making the metal less available for pathogens. This process, termed nutritional immunity, is mediated by various cytokines and hepcidin, whose expression gets upregulated upon multiple inflammatory and danger signals (9). Such alterations of iron homeostasis also occur in association with other inflammatory processes including cancer (10) characterized by normal or high iron stores as reflected by increased levels of ferritin whereas circulating iron levels and saturation of transferrin with iron are low. This functional iron deficiency causes iron limitation of erythroid progenitor cells and contributes to the development of so called anemia of inflammation (AI) or anemia of chronic disease (ACD) or anemia of cancer (11). In addition, this also limits iron availability for cancer but also for immune cells such as lymphocytes and may thus impact on anti-cancer immune effector function and even on the efficacy of anti-tumor immunotherapy. There is evidence from literature that this can be traced back to effects of iron on immune and cancer cell proliferation and differentiation, innate immune function and regulation of cellular metabolic processes including mitochondrial activity and micro RNA processing (10, 12–15).

Breast cancer is the most common type of cancer in women worldwide and, despite the enormous progress in diagnosis and treatment, it still represents one of the main causes of cancer-related death. Several studies have shown a link between dysregulation of iron metabolism and progression of breast cancer (16, 17). Particularly, spatio-temporal accumulation of iron in the tumor-microenvironment was linked to an increased cancer risk and poor outcome, respectively (18, 19). Mechanistically, apart from the effects of iron on immune function, the metal can stimulate cancer metabolism, alter iron dependent redox balance, which increases mutation rates, organelle damage, loss of tumor suppressors, oncogene expression and triggers pro-oncogenic signaling like Wnt and NFκB pathways (20–22).

Tumor growth and progression can be both enhanced and inhibited by cells of the immune system including T cells by a process which is called immunosurveillance (23). T lymphocytes as components of the adaptive immune system can destroy tumor cells *in situ*. The predominant tumor infiltrating lymphocytes are CD4^+^ T helper cells, CD8^+^ cytotoxic T cells, and regulatory T cells (24). CD4^+^ T cells are classified into T_H_1 cells secreting proinflammatory cytokines like IFNγ and IL-2, whereas T_H_2 cells secrete IL-4, IL-5, IL-10, and IL-13. T_H_2 cytokines induce T cell anergy and lead to an increase of humoral B cell function (25, 26). The primary role of CD4^+^ helper T cells in tumor response is to assist in the activation of CD8^+^ T cell mediated cell killing. Most tumor cells are positive for MHC class I, but negative for MHC class II, which makes the primary anti-tumor response dependent on CD8^+^ cytotoxic T cells (27). In cancer patients a tumor response involving CD8^+^ T cells, T_H_1 CD4^+^ T cells, and IFNγ producing natural killer cells is associated with a better prognosis (28). In contrast, a B cell and T_H_2 polarized response can promote tumor development and progression (28). Immunosuppressive effects of iron on the T cell response have been described. Iron can trigger CD4^+^ differentiation towards a T_H_2 phenotype (14, 29) and impact on CD8^+^ cell numbers (30). A similar impairment of T cell function has been observed in individuals with hereditary or transfusion mediated iron overload (31, 32).

Of note, individuals carrying the homozygous *HFE* C282Y mutation, the most common cause for hereditary hemochromatosis, are at increased risk of developing cancer, including breast cancer (19). Whether this is a direct consequence of iron toxicity or related to quantitative or qualitative alterations in T cell subsets remains unknown (33).

In spite of the direct effects of iron on tumor cells and anti-tumor immunity, the impact of intravenous iron preparations used for treatment of cancer related anemia towards the further clinical course and outcome of cancer along with their impact on specific cancer therapy is still unknown (34, 35). On the one hand, the functional iron deficiency caused by tumor-accompanying inflammation may be regarded as a measure to limit tumor progression, on the other hand, iron deficiency and ACD may result in suboptimal delivery of iron needed for immune cell function.

Herein we demonstrate that isomaltosoide, an iron formulation used for correction of iron deficiency in humans, negatively impacts on the efficacy of cancer immunotherapy and combined IL-2/doxorubicin chemo-immunotherapy in a murine E0771 mammary carcinoma model. *In vivo*, iron supplementation led to accelerated cancer growth and impaired efficacy of the investigated therapy protocols along with diminished tumor infiltration by cellular effectors of anti-tumor response, T_H_1 and cytotoxic T cells. Mechanistically we show that iron, both in transferrin-bound and non-transferrin bound form, dramatically brakes CD4^+^ and CD8^+^ T cell proliferation and cytokine production and promotes cell death.

## Material and Methods

### Cell Line

E0771 mouse adenocarcinoma cells (obtained from ATCC) were maintained in DMEM (Dulbecco`s Modified Eagles`s Medium; PAN Biotech) plus 10% fetal calf serum (FCS; Biochrom) plus 1% penicillin/streptomycin (Lonza) plus 2 mM L-glutamine (Lonza) at 37°C, plus 5% carbon dioxide (36).

### Mice

Female C57Bl/6N mice (obtained from Charles River) had free access to food and water and were housed according to institutional and governmental guidelines in the animal facility of the Medical University of Innsbruck with a 12-hour light-dark cycle and an average temperature of 20°C ± 1°C. Animals were kept on a standard rodent diet (SNIFF, Soest, Germany). Blood was taken through the facial vein and blood counts were measured with a VetABC Animal Blood Counter. Animal experiments were approved by the Austrian Federal Ministry of Science and Research (BMWF-66.011/0117-WF/V/3b/2017) according to the directive 2010/63/EU.

### Implantation of Tumors

C57Bl/6 derived E0771 adenocarcinoma cells were washed twice in PBS and 2.5 × 10^5^ cells injected into one of the inguinal mammary glands into 8–12 weeks old female C57Bl/6N mice under short-term inhalation anesthesia with isoflurane. Three days after tumor implantation mice were given intravenously 2 mg elementary iron in the form of iron isomaltoside (Monofer; Pharmacosmos) or PBS. Tumor growth was monitored weekly by caliper measurements of length (l) and width (w). Tumor volume was calculated with the formula V = lw^2^π/6. Three weeks after tumor implantation mice were sacrificed by cervical dislocation, and tumors were isolated by surgical excision.

### Tumor Therapy

For checkpoint immunotherapy, tumor-bearing mice were intraperitoneally administered anti-mouse PD-L1 antibodies (0.5 mg/animal, clone10F.9G2; BioXCell) every third day starting from day 1 after tumor implantation. For chemo-immunotherapy, doxorubicin (5 mg/kg, Accord) was administered intraperitoneally into tumor-bearing mice once on day eight after tumor implantation and recombinant murine IL-2 (100,000 IU per animal, Peprotech) daily starting on day nine after tumor implantation (37).

### Isolation of Tumor-Infiltrating Lymphocytes

Tumor tissue was minced and digested with Liberase TM (0.15 Wünsch-Units/ml, Roche) and 10 µg/ml DNaseI (Roche) in FCS-free RPMI-1640 (PAN Biotech) medium with constant mixing (250 rpm), at 37°C for 1 h. Tumor cell suspension was collected through a 100 µm cell strainer into a tube containing RPMI-1640 (PAN Biotech) plus 10% FCS (Biochrom) plus 1% penicillin/streptomycin (Lonza) plus 2 mM glutamine (Lonza) and centrifuged at 300*g* for 5 min. Red blood cells were lysed by incubation in ACK buffer (150 mM NH_4_Cl, 10 mM KHCO_3_, 0.1 mM Na_2_EDTA) for 2 min at room temperature. Cell suspension was filtered through a 40 µm cell strainer and used for flow cytometry staining.

### Flow Cytometry Analysis

Flow cytometry staining was performed with panels of antibodies specific for activated/memory T cells (αCD3-Biotin, αCD4-FITC, αCD8-APCeF780, αCD62L-PeCy7, αCD44-APC; all from BioLegend) in PBS with 0.5% FCS 2 mM EDTA for 15 min. For intracellular staining cells will be stimulated with a mix containing 10 µg/ml Brefeldin A (Sigma), 50 ng/ml PDBu (Sigma) and 500 ng/ml ionomycin (Sigma) in RPMI-1640 (PAN Biotech) plus 10% FCS (Biochrom) plus 1% penicillin/streptomycin (Lonza) plus 2 mM L-glutamine (Lonza) for 4 h. The cells were then formalin-fixed, permeabilized (0.05% Triton X-100 in PBS) and stained for cytokines (αIL-2-PE, αIFNγ-PeCy7), and transcription factors (αFOXP3-FITC) or perforin (αPerforin-APC) for 1 h. All antibodies were from Biolegend. Cells were analyzed with Gallios and Cytoflex S flow cytometers (Beckman Coulter) and FlowJo Software (Beckton Dickinson).

### Splenocyte Cell Culture

Spleens were isolated from tumor-naive female C57Bl/6N mice. After lysis of erythrocytes using the Mouse Erythrocyte Lysing Kit (R&D Systems) 2.5 × 10^5^ splenocytes per well were then seeded in a 96-well round bottom plate and stimulated with 4 µg/ml plate-bound or 1 µg/ml soluble rat anti-mouse CD3 (clone 17A2; BD Pharmingen). Ferric chloride FeCl_3_ (Sigma Aldrich), ferric sulfate Fe_2_(SO_4_)_3_ (Sigma Aldrich), ferric citrate FeC_6_H_5_O_7_ (Sigma Aldrich), and holo-transferrin were added at concentrations of 2.5µM, 5 µM, 10 µM and 20 µM elementary iron. Splenocytes were cultured in RPMI-1640 medium (PAN Biotech) supplemented with 10% FCS (Biochrom), 2% sodium pyruvate (Sigma), 1× non-essential amino acids (Gibco), 0.01% β-mercaptoethanol (Roth), 1% penicillin/streptomycin (Lonza) and 2 mM L-glutamine (Lonza).

### BrdU Labeling of Splenocytes

Splenocytes were cultured as described before and pulsed with 10 µM BrdU (Sigma-Aldrich) 4 h before harvesting. Intracellular staining for BrDU with surface co-staining for CD3, CD4 and CD8 was performed with BrdU Flow Kit (BD) according to the manufacturers` instructions and cells were analyzed with flow cytometry. Iron sources ferric chloride FeCl_3_, ferric sulfate Fe_2_(SO_4_)_3_, ferric citrate FeC_6_H_5_O_7_, and holo-transferrin were added at indicated concentrations.

### CFSE Labeling of Splenocytes

Before culture splenocytes were washed twice with PBS and labeled with 2.5 µM CFSE (eBioscience) in PBS for 10 min at 37°C, followed by a wash with RPMI-1640 (PAN Biotech) supplemented with 10% FCS (Biochrom). CFSE dilution after 96 h of culture was measured with flow cytometry. Where indicated, the ferroptosis inhibitor Ferrostatin-1 (1 µM, Sigma), cytoplasmic ROS scavenger NAC (N-acetylocysteine, 10 mM, Sigma), necroptosis inhibitor Necrostatin-1 (30 µM, Sigma), mitochondrial ROS scavenger MitoTEMPO (20 µM, Sigma), or the caspsase-3-inhibitor z-DEVD-FMK (20 µM, BD) were added. Iron was added in the form of 5 µM ferric citrate.

### Iron Measurement

Tissue iron was quantified using a colorimetric method with bathophenanthroline disulfonic acid (38). In brief, organ lysates were hydrolyzed with acid for 24 h at 65°C, mixed with a colorimetric solution containing sodium acetate, bathophenanthroline disulfonic acid and l-ascorbic acid and absorbance at 539 nm was measured. The iron content of the organ was calculated from a standard curve and normalized to the protein content of the lysate determined by the Bradford method.

### ROS Measurement

Splenocytes were cultured as described before. For the determination of mitochondrial and cytoplasmic ROS, cells were stained with 2.5 µM MitoSOX (Thermofisher) and 2.5 µM DCFDA (Sigma), respectively, for 30 min at 37°C and analyzed by flow cytometry. Splenocytes stimulated for 4 h with the inhibitor of mitochondrial oxidative phosphorylation rotenone (2.5 µM, Sigma) served as a positive control for cytoplasmic and mitochondrial ROS.

### Statistics

Statistical analysis was performed with GraphPad Prism 7 and R programming suite (version 3.6.3) with a tidyverse package bundle and ggplot2 graphics library. If not stated otherwise, data are plotted as mean with SEM presented as bars and whiskers and single animals/observations presented as points or symbols. Normality of variable distribution was assessed by Shapiro–Wilk test and visual inspection of the quantile–quantile plots. Statistical significance for two-group comparisons was determined by a two-tailed T-test for normally distributed variables and by the Mann–Whitney U test for non-normally distributed variables. Statistical significance for comparisons of more groups/factors was analyzed by one- or two-way ANOVA, as appropriate, with Tuckey post-hoc test.

**Figure 1:**
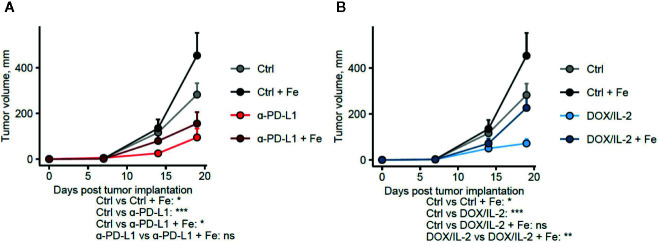
Administration of iron negatively influences the efficacy of different immunotherapies. Female C57Bl/6 mice were subcutaneously implanted with E0771 cells (2.5 × 10^5^ cells per animal), supplemented with intravenous iron isomaltoside (Fe, 2 mg elementary iron per animal) 3 days after tumor implantation and treated with anti-PD-L1 **(A)** or IL-2 and doxorubicin **(B)** as described in *Materials and Methods*. Therapy-naive: n = 17, therapy-naive/iron: n = 5, anti-PD-L1: n = 14, anti-PD-L1/iron: n = 17, IL-2/doxorubicin: n = 13, IL-2/doxorubicin/iron: n = 14. Tumor volume was determined weekly by caliper measurements. Statistical significance was determined by mixed-effect multiple linear regression (fixed effects: time point and time point: treatment group interaction, random effect: individual animal). Group means with SEM are presented. P values were corrected for multiple comparisons with Benjamini–Hochberg method. P values for differences in growth rate between the untreated control and the given group and for the differences in growth rate between the immunotherapy and immunotherapy/iron groups (the time point: treatment group interaction term estimates) are presented under the plots. ns: not significant, *p < 0.05, **p < 0.01, ***p < 0.001.

Differences in tumor growth rate (**Figure 1**) (1) between the untreated tumor bearers and treatment groups (iron alone, immunotherapy alone, and iron with immunotherapy) and (2) between the immunotherapy- and immunotherapy/iron-treated animals were analyzed with separate mixed-effect multiple linear regression models (fixed effects: time point and therapy group: time point interaction, random effect: individual animal, R packages lme4 and lmer test). Regression estimates for the therapy group: time point interaction term was assumed to model differences in tumor growth rate (1) between the untreated animals and the respective therapy regimen and (2) between the immunotherapy and immunotherapy/iron group.

Statistical significance for differences in T cell counts in cultures stimulated with iron, ROS scavengers or inhibitors of cell death (**Figure 8**) was determined with mixed-effect linear modeling (fixed effects: iron, cell death/ROS inhibitor and the iron: cell death/ROS inhibitor interaction; random effect: cell donor). The estimate of the iron: cell death/ROS inhibitor interaction term was deemed the measure of reversal of iron effects on T cell expansion.

In linear modeling, statistical significance for the regression estimates was determined by a two-tailed T-test (estimate ≠ 0; degrees of freedom calculated with Satterthwaite formula, package ImerTest) and corrected for multiple comparisons with the Benjamini–Hochberg method.

**Figure 2:**
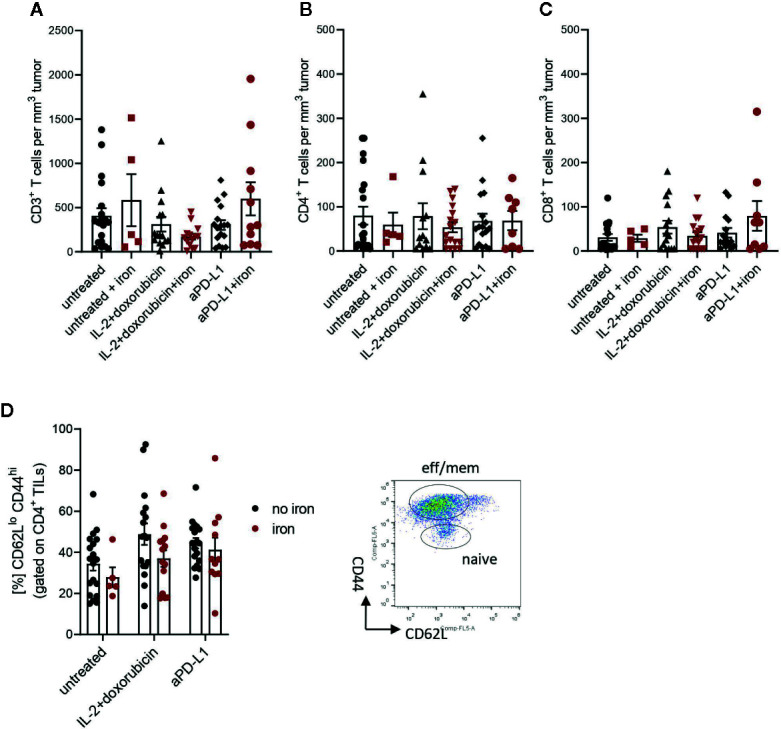
Administration of intravenous iron has no influence on the numbers of effector CD3^+^ **(A)**, CD4^+^ **(B)**, CD8^+^ **(C)** and effector-memory **(D)** tumor infiltrating lymphocytes in different immunotherapeutic settings. Naive TILs were identified as CD62L^hi^CD44^lo^, effector-memory TILs were described as CD62L^lo^CD44^hi^ in tumors 21 days post implantation. Mean with SEM is presented in the plots. Statistical significance was determined by 2-way ANOVA. untreated n = 20, untreated + iron n = 5, IL-2 + doxorubicin n = 15, IL-2 + doxorubicin + iron n = 14, aPD-L1 n = 17, aPD-L1 + iron n = 11. The results of ANOVA are presented in *Materials and Methods*/Specific statistical data analysed in main figures.

**Figure 3:**
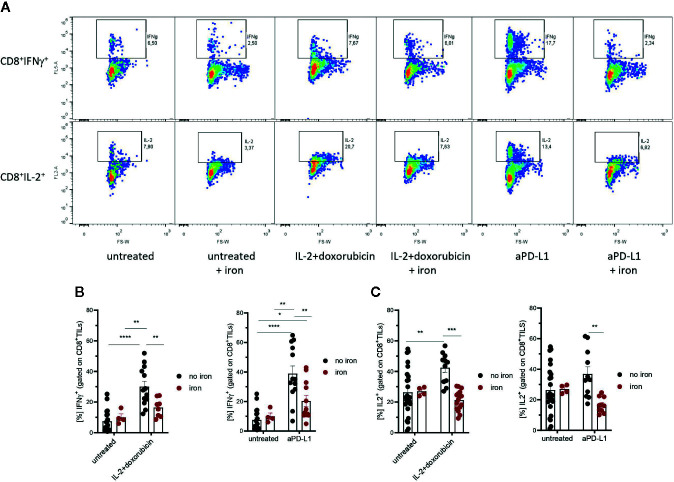
Effects of intravenous iron on functional T cell subsets. Intravenous iron supplementation significantly reduces the function of CD8^+^ tumor infiltrating lymphocytes **(A**–**C)**. Representative plots are shown (mean ± SEM). Statistical significance was determined by 2-way ANOVA. The results of Tuckey post-hoc-test are presented in the plots: ns: not significant, *p < 0.05, ** p < 0.01, ***p < 0.001, ****p < 0.0001. untreated n = 20, untreated + iron n = 4, IL-2 + doxorubicin n = 13, IL-2 + doxorubicin + iron n = 8, aPD-L1 n = 13, aPD-L1 + iron n = 12. The results of ANOVA are presented in *Materials and Methods*/Specific statistical data analyzed in main figures.

**Figure 4:**
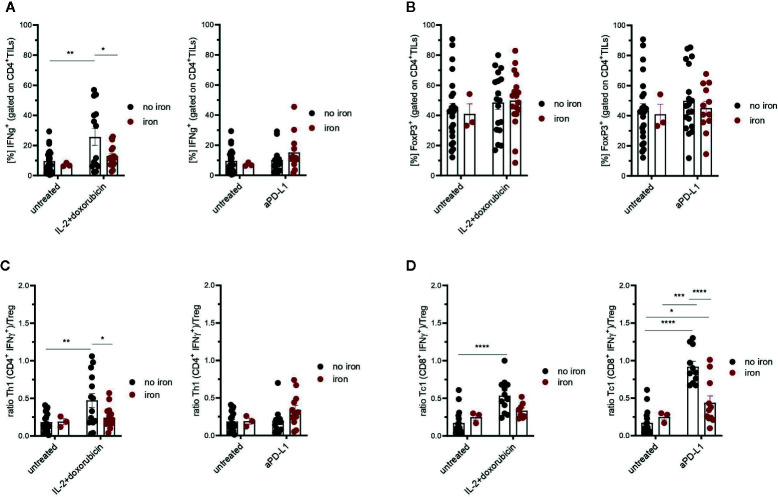
Effects of intravenous iron on CD4^+^ TILs **(A)**, regulatory T cells **(B)** and Th1/Treg or Tc1/Treg ratios **(C, D)** in immunotherapy and chemoimmunotherapy of mouse mammary carcinomas. Ratios Th1/Treg **(C)** and Tc1/Treg **(D)** were calculated. Representative plots are shown (mean ± SEM). Statistical significance was determined by 2-way ANOVA. The results of Tuckey post-hoc-test are presented in the plots: ns: not significant, *p < 0.05, **p < 0.01, ***p < 0.001, ****p < 0.0001. untreated n = 24, untreated + iron n = 3, IL-2 + doxorubicin n = 14, IL-2 + doxorubicin + iron n = 17, aPD-L1 n = 13, aPD-L1 + iron n = 12. The results of ANOVA are presented in *Materials and Methods*/Specific statistical data analyzed in main figures.

**Figure 5:**
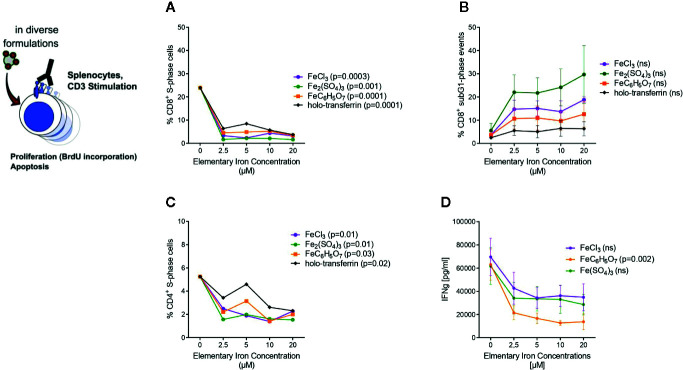
Transferrin bound and non-transferrin bound iron impairs T cells proliferation and promotes apoptosis. Splenocytes isolated from tumor-naive C57Bl/6N mice were stimulated with plate-bound anti-CD3 antibodies and supplemented with iron in the form of holo-transferrin (transferrin bound iron, TBI), ferric chloride FeCl_3_, ferric sulfate Fe_2_(SO_4_)_3_, or ferric citrate FeC_6_H_5_O_7_ (non-transferrin bound iron, NTBI). BrdU incorporation and cell cycle distribution in CD4^+^ and CD8^+^ T cells was measured by flow cytometry **(A**–**C)** and IFNγ concentration in culture supernatant was determined by Multiplex 72 h after culture start **(D)**. Statistical significance was assessed by one-way ANOVA for each iron source. Each point represents mean with SEM from n = 3 independent experiments.

**Figure 6:**
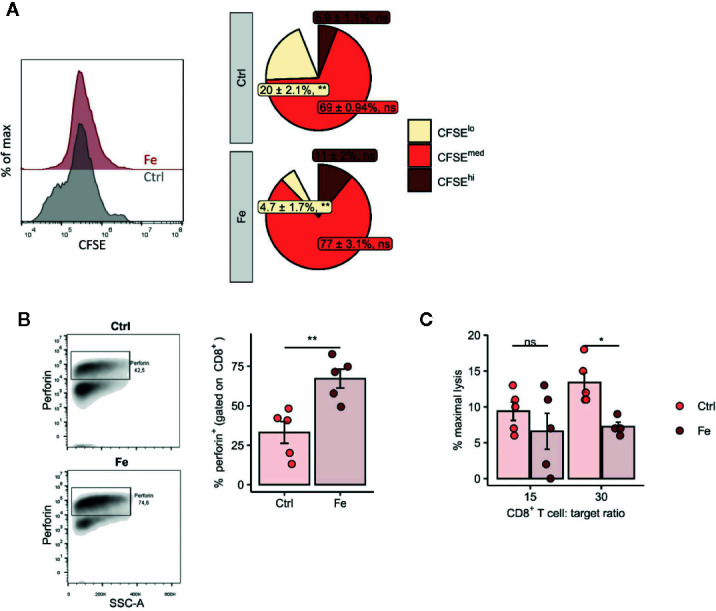
*In vitro* addition of iron to splenocytes decreases the number of proliferating CD8^+^ T cells (CFSE low) **(A)**, negatively affects perforin degranulation in CD8^+^ cytotoxic T cells **(B)** and significantly reduces the CD8^+^ T cell dependent lysis of target cells **(C)**. **(A, B)** Splenocytes isolated from tumor-naive C57Bl/6N mice were stimulated with plate-bound anti-CD3 antibodies and iron in form of iron citrate (FeC_6_H_5_O_7_; non-transferrin bound iron, NTBI) was added. Proliferation of CD8^+^ T cells was measured by flow cytometry depending on CFSE 72h after culture start. Data are presented as Pie Plots (mean ± SEM) n = 4. Perforin was stained intracellularly as described in *Materials and Methods* n=5. Statistical significance was determined by a two-tailed T-test and corrected for multiple comparisons with the Benjamini–Hochberg method. **(C)** The capability of iron treated and non-iron treated CD8^+^ T cells to lyse target cells was measured with a chromium release assay as described in *Material and Methods*. Representative flow cytometry results and summary plots are shown (mean ± SEM). Statistical significance was determined by 2-way ANOVA. The results of Tuckey post-hoc-test are presented in the plots: ns: not significant, *p < 0.05, **: p < 0.01. control, Fe (ratio 15:1) n = 5, control, Fe (ratio 30:1) n = 4. The results of ANOVA are presented in *Materials and Methods*/Specific statistical data analyzed in main figures.

**Figure 7:**
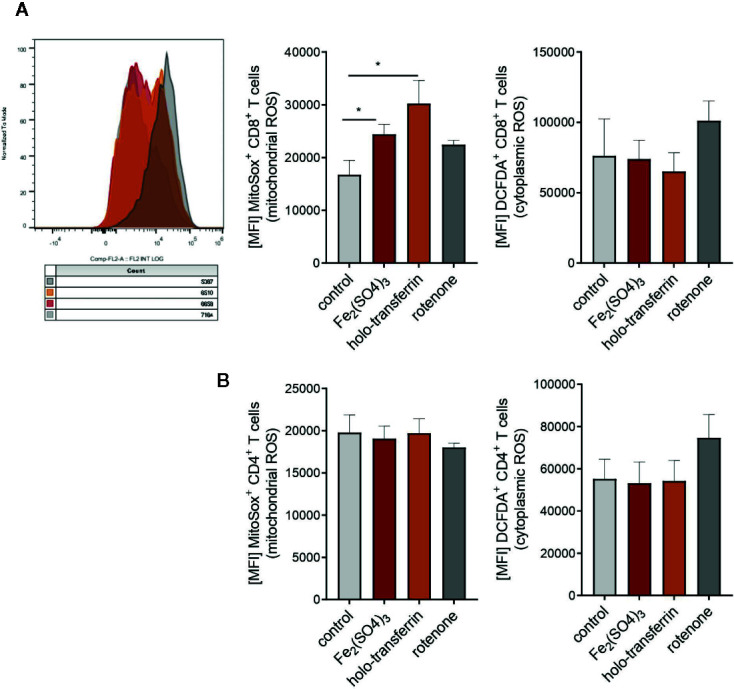
Iron administration to splenocytes leads to oxidative stress and increased production of mitochondrial reactive oxygen species (ROS). Splenocytes were isolated from tumor-naive C57Bl/6N female mice and cultured in 96 well plates coated with anti-CD3. Fe_2_(SO_4_)_3_ and holo-transferrin were added as NTBI and TBI, the inhibitor of oxidative phosphorylation rotenone was used as a positive control for ROS formation. After 24h DCFDA^+^ and MitoSox^+^ CD8^+^ T cells **(A)** and CD4^+^ T cells **(B)** were analysed by flow cytometry. DCFDA is defined as indicator for cytoplasmic ROS, MitoSox for mitochondrial ROS. Statistical significance was determined by Student`s t-test. Representative flow cytometry results and summary plots are shown (mean±SEM; n = 3).

**Figure 8:**
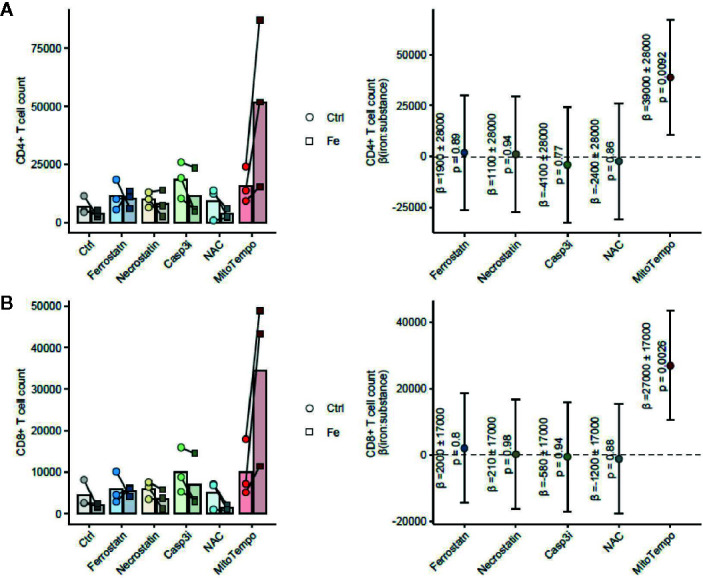
The mitochondrial ROS scavenger MitoTempo reverses the iron-mediated inhibition of T cell growth. Splenocytes were isolated from tumor-naive C57Bl/6 mice (n = 3 separate cell donors) and cultured for 72 h in presence of 1 µg/ml activating anti-CD3 antibody and the inhibitors of ferroptosis (Ferrostatin: 1 µM), necroptosis (Necrostatin: 30 µM), apoptosis (Casp3i, z-DEVD-FMK: 20 µM) or cytoplasmic (NAC, N-acetylcysteine, 10 mM) or mitochondrial (MitoTempo, 20 µM) ROS scavengers. CD4^+^ T cells **(A)** and CD8^+^ T cells **(B)** were enumerated by flow cytometry. Statistical significance for reversal of the iron-mediated inhibition of T cell growth measured as the positive interaction of iron and cell death/ROS inhibitor was assessed by mixed-effect linear regression (fixed effects: iron, cell death/ROS inhibitor and the iron: cell death/ROS inhibitor interaction; random effect: cell donor). Left panels: cell counts are presented as points, lines connect data for the same cell donor; right panels: forest plots showing the regression coefficients (beta) of the iron:cell death/ROS inhibitor interaction as points and 95% confidence intervals as error bars.

Specific statistical data analyzed in main figures:

**Figures 1A, B**: Mixed-effect multiple linear regression (fixed effects: therapy group and therapy group: timepoint interaction, random effect: individual animal). P values for the time:therapy interaction model terms are shown in the plots.
**Figure 2**: Two-way ANOVA (**A**), (**B**), (**C**), (**D**) ns.
**Figure 3B**: 2-way ANOVA: treatment IL-2 + doxo, F_(1, 41)_ = 22 P <0.0001; iron, F_(1, 41)_ = 3.2 ns; treatment:iron interaction, F_(1, 41)_ = 6.9, P = 0.012; treatment aPD-L1, F_(1, 45)_ = 25, P <0.0001; iron, F_(1, 45)_ = 3.5, ns; treatment:iron interaction, F_(1, 45)_ = 6.2, P = 0.016; Tukey`s post test results presented in the plots.
**Figure 3C**: 2-way ANOVA: treatment IL-2 + doxo, F_(1, 52)_ = 1.7, ns; iron, F_(1, 52)_ = 6.6, P = 0.013; treatment:iron interaction, F_(1, 52)_ = 7.5, P = 0.0086; treatment aPD-L1, F_(1, 46)_ = 0.00030, ns; iron, F_(1, 46)_ = 4.3, P = 0.044; treatment:iron interaction, F_(1, 46)_ = 4.9, P = 0.032; Tukey`s post test results presented in the plots.
**Figure 4A**: 2-way ANOVA: treatment IL-2 + doxo, F_(1, 54)_ = 6.55, P = 0.014; iron, F_(1, 54)_ = 3.1, ns; treatment:iron interaction, F_(1, 54)_ = 1.4, ns; treatment aPD-L1, F_(1, 49)_ = 1.8, ns; iron, F_(1, 49)_ = 1.2, ns; treatment:iron interaction, F_(1, 49)_ = 1.3, ns; Tukey`s post test results presented in the plots.
**Figure 4B**: 2-way ANOVA: treatment IL-2 + doxo, F_(1, 59)_ = 0.96, ns; iron, F_(1, 59)_ = 0.0091, ns; treatment:iron interaction, F_(1, 59)_ = 0.080, ns; treatment aPD-L1, F_(1, 54)_ = 5.5, ns; iron, F_(1, 54)_ = 0.27, ns; treatment:iron interaction, F_(1, 54)_ = 0.024, ns; Tukey`s post test results presented in the plots.
**Figure 4C**: 2-way ANOVA: treatment IL-2 + doxo, F_(1, 50)_ = 5.0, P = 0.030; iron, F_(1, 50)_ = 2.2, ns; treatment:iron interaction, F_(1, 50)_ = 2.4, ns; treatment aPD-L1, F_(1, 45)_ = 1.9, ns; iron, F_(1, 45)_ = 1.6, ns; treatment:iron interaction, F_(1, 45)_ = 1.4 ns; Tukey`s post test results presented in the plots.
**Figure 4D**: 2-way ANOVA: treatment IL-2 + doxo, F_(1, 36)_ = 12, P = 0.0017; iron, F_(1, 36)_ = 0.80, ns; treatment:iron interaction, F_(1, 36)_ = 4.4, P = 0.042; treatment aPD-L1, F_(1, 38)_ = 30, P <0.0001; iron, F_(1, 38)_ = 5.5, ns; treatment:iron interaction, F_(1, 38)_ = 11, P = 0.0022; Tukey`s post test results presented in the plots.
**Figure 5**: one-way ANOVA for particular iron forms, ANOVA p values presented in the plot legends.
**Figure 6A**: two-tailed T test to compare the percentages of CFSE^hi^, CFSE^med^ and CFSE^lo^ cells between control- and iron-stimulated cultures, corrected for multiple comparisons with Benjamini–Hochberg method. P values presented in the pie plot.
**Figure 6B**: two-tailed T test, p values presented in the plot.
**Figure 6C**: two-way ANOVA: iron, F_(1, 15)_ = 7.2, p = 0.017; T cell–target ratio, F_(1, 15)_ = 2.1, ns; iron: T cell–target ratio interaction, F_(1, 15)_ = 0.99, ns; Tukey`s post test results presented in the plots.
**Figure 7**: Two-tailed T test for control–iron comparisons, p values presented in the plots.
**Figure 8**: Mixed-effect multiple linear regression (fixed effects: fixed effects: iron, cell death/ROS inhibitor and the iron: cell death/ROS inhibitor interaction, random effect: cell donor). P values for the iron: cell death/ROS inhibitor interaction interaction model terms are shown in the Forest plots.

Chromium release assay: Murine B16/OVA melanoma cells as target cells were cultivated in DMEM medium (PAN Biotech) supplemented with 10% FCS (Biochrom), 1% penicillin/streptomycin (Lonza) and 2 mM L-glutamine (Lonza). The assay was performed as described (39). Briefly, 2 × 10^6^ target cells were labeled with 200 µCi Na_2_Cr^51^O_4_ (specific activity 300 to 500 Ci/g chromate; Hartmann Analytik) for 1 h at 37°C, washed once, and resuspended at a concentration of 5 × 10^4^/ml in medium. As effector cells CD8^+^ T cells were isolated from spleens of C57BL/6-Tg(TcraTcrb) 1,100 Mjb/Crl mice (OTI mice) with the help of the MagniSort Mouse CD8 T cell Kit (Thermo Fisher Scientific). 2.5 × 10^6^ cells/ml were then seeded in a 96 well U-bottom plate (Falcon) and stimulated with 1 µg/ml soluble rat anti-mouse CD3 (clone 17A2; BD Pharmingen) and 1 µg/ml hamster anti-mouseCD28 (clone 37.51; BD Pharmingen) in RPMI-1640 medium (PAN Biotech) supplemented with 10% FCS (Biochrom), 2% sodium pyruvate (Sigma), 1× non-essential aminoacids (Gibco), 0.01% β-mercaptoethanol (Roth), 1% penicillin/streptomycin (Lonza) and 2 mM L-glutamine (Lonza). After 24 h CD8^+^ T cells were primed for 1 h with 1 µg/ml OVA (257–264) (Anaspec). About 20 µl of target cells (5 × 10^3^) were incubated with 200 µl of various amounts of effector cells with effector:target (E:T) ratios ranging from 30:1 to 7.5:1. After 4 h of incubation in a humidified 5% CO_2_/95% air atmosphere, 100 µl of the culture supernatant were counted with a gamma-scintillation counter. Results are presented as percentage of specific lysis.

## Results

The administration of intravenous iron is an established therapy for cancer-related anemia but its effects on the underlying malignancy, anti-tumor immunity and efficacy of tumor immunotherapy remain incompletely understood. We thus investigated the effects of intravenous administration of a clinically applicable iron preparation, ferric isomaltoside, in the implantable E0771 mouse mammary carcinoma model. Of note, mice bearing E0771 neoplasms display mild impairment of erythropoiesis as demonstrated by a significantly reduced blood hemoglobin content and hematocrit as compared with tumor-free mice (**Supplementary Figure 1**), hence, in part, mimicking cancer-anemia phenotype observed at a substantial percent of breast cancer patients.

Iron concentrations used for *in vivo* iron studies in mice differ a lot (0.27–35 mg per mouse) and furthermore the basal metabolic rate per gram body weight in mice is higher than in humans (40). Therefore, we used a supra-clinical dose of 2 mg per mouse (approx. 100 mg/kg, corresponding to 6–8 g in humans), which was found to cause a significant accumulation of iron in the canonical iron-storage organs, spleen and liver, in tumor-free animals (spleen P = 0.033; liver P = 0.0013, **Supplementary Figures 2A, B**). To investigate, if such iron supplementation may cause a similar iron accumulation in the tumor tissue, we implanted E0771 adenocarcinoma cells into wildtype C57Bl/6N female hosts followed by intravenous administration of ferric isomaltoside 3 days after tumor implantation. As shown in **Supplementary Figure 2C** **,** we could not observe any increase of tissue iron measured with the colorimetric, bathophenanthroline disulfonic acid-based assay in the neoplastic tissue on day 21 post implantation arguing against an overt iron overload in the tumor like in the liver and spleen. mRNA levels of transferrin receptor 1 (TFR1 or CD71) are tightly negatively regulated by biologically active intracellular iron (41) and, thus, cell surface levels of the protein may be used as a sensitive surrogate marker for gauging iron availability in the tumor microenvironment. Interestingly, both CD45^-^ tumor epithelial cells as well as CD45^+^ tumor-infiltrating leukocytes isolated from the iron-treated E0771 tumor mice demonstrated significantly decreased cell surface levels of CD71 (tumor epithelium and leukocytes) and percentages of CD71-positive cells (tumor epithelium) indicative of a better availability of reactive iron in the tumor milieu upon systemic intravenous iron supplementation (**Supplementary Figure 2D**).Our data indicate that intravenous iron accumulates in the spleen and liver without altering the total iron content of the tumor tissue and increasing the local intracellular availability of reactive iron in the malignant tissue.

As a therapy, mice were either treated with immunotherapy in the form of repeated anti-PD-L1 antibody injections every third day, starting at tumor implantation, or chemo-immunotherapy in the form of single doxorubicin injection followed by daily administration of highly dosed IL-2 starting on day 8 after tumor implantation.

Checkpoint immunotherapy with anti-PD-L1 or combined chemo-immunotherapy with IL-2 and doxorubicin significantly reduced tumor growth as compared with therapy-naive mice (p = 0.00031 and p = 0.00011, respectively), whereas iron supplementation without any therapy led to a significantly faster tumor progression (p = 0.039) for comparison with therapy- and iron-naïve animals. In addition, intravenous iron supplementation led to a substantial albeit not significant reduction of the therapeutic effects of checkpoint anti-PD-L1 therapy (**Figure 1A** **,** p = 0.016 for the therapy naive–anti-PD-L1/iron group comparison and p = 0.10 for the anti-PD-L1–anti-PD-L1/iron group comparison) and to a significant reduction of the efficacy of IL-2/doxorubicin therapy (**Figure 1B**, p = 0.15 for the therapy naive–IL-2/doxorubicin/iron group comparison and p = 0.0032 for the IL-2/doxorubicin–IL-2/doxorubicin/iron group comparison). We then studied whether the impaired therapeutic effect of both therapies upon iron loading is linked to the function of tumor infiltrating lymphocytes, such as CD8^+^ cytotoxic T cells (Tc1), which are the responsible subset for effective anti-tumor T cell response, and/or CD4^+^ T helper cells which are needed as assist to ensure full functionality of CD8^+^ cytotoxic T cells.

Therefore, tumor infiltrating T cells were isolated. Interestingly, we could not detect any significant, iron- or cancer therapy-dependent differences in the numbers of CD3^+^, CD4^+^ and CD8^+^ tumor infiltrating lymphocytes per mm^3^ tumor (**Figures 2A–C** respectively). Although the percentage of CD4^+^ effector-memory cells (CD4^+^CD44^hi^CD62L^lo^) was consistently reduced in mice receiving intravenous iron, these changes were not statistically significant (**Figure 2D**). However, when we further studied the function of tumor infiltrating lymphocytes, we found that intravenous iron supplementation significantly reduced the production of cytokines IL-2 and IFNγ by tumor CD8^+^ cytotoxic T cells, indicating iron-dependent reduced functionality of these cells (**Figures 3A, B, C**). Of note, also CD4^+^ T helper cells in our tumor model showed reduced functionality as reflected by reduced production of IFNγ. However, this effect could only be demonstrated in IL-2/doxorubicin treatment (**Figure 4A**). Of interest, we could not find any differences in the percentage of regulatory T cells (CD4^+^FoxP3^+^) (**Figure 4B**). Although T_H_1/Treg ratios were found significantly lowered by iron solely for the IL-2/doxorubicin protocols, the highly significantly diminished Tc1/Treg ratios upon iron supplementation could be observed for both treatments, most of all in anti-PD-L1 treatment (**Figures 4C, D**).

Based on these observations, we asked whether iron generally influences proliferation and cytokine production of CD8^+^ cytotoxic T cells and CD4^+^ T helper cells. In the body fluids, iron can generally exist in two forms: as transferrin-bound iron (TBI), when iron concentration does not exceed binding capacities of transferrin, and as chemically reactive, potentially toxic non-transferrin-bound iron (NTBI), when the concentration of iron is higher than the binding capacity of transferrin (42). Importantly, both forms can be taken up by T cells (43, 44). We isolated splenocytes from tumor-naive C57Bl/6N female mice and stimulated them with anti-CD3 antibodies. To induce NTBI, we supplemented the culture with 5 µM ferric iron, a concentration shown by us and others to generate measurable NTBI (43, 45), in the form of salts ferric chloride (FeCl_3_), ferric sulfate (Fe_2_(SO_4_)_3_), and ferric citrate (FeC_6_H_5_O_7_). Holo-transferrin was added as a source of TBI. Iron, both in its physiological TBI form as well as NTBI, halted proliferation of both CD8^+^ cytotoxic T cells and CD4^+^ T helper cells as shown by a dramatically reduced fraction of S-phase cells (CD8^+^ S-phase cells FeCl_3_ p = 0.0003, Fe_2_(SO_4_)_3_ p = 0.001, FeC_6_H_5_O_7_ p = 0.0001, holo-transferrin p = 0.0001; CD4^+^ S-phase cells FeCl_3_ p = 0.01, Fe_2_(SO_4_)_3_ p = 0.01, FeC_6_H_5_O_7_ p = 0.03, holo-transferrin p = 0.02 and promoted apoptosis measured by sub-G1 fractions (**Figures 5A–C**). This phenomenon was paralleled by a strongly decreased production of the key anti-tumor cytokine IFNγ by CD4^+^ cells (FeC_6_H_5_O_7_ p = 0.002) (**Figure 5D**). Importantly, the detrimental effects of iron on T cell expansion were corroborated by the results of another proliferation assay employing dilution of the fluorescent CFSE dye (CFSE low population p = 0.0055) (**Figure 6A**). CFSE is a widely used method to monitor lymphocyte proliferation due to the progressive halving of CFSE fluorescence within daughter cells following each cell division (46).

Following these observations, we tested the effect of iron on the cellular levels on the turnover of the cytolytic protein perforin in *in vitro* iron or non-iron supplemented splenocytes. Perforin is found in the granules of CD8^+^ cytotoxic T cells and is centrally involved in anti-cancer immune function whereby perforin binds to the cell membrane of target cells, forming a pore allowing for granzyme B injection and killing of the target cell (47). We found increased intracellular perforin in iron-stimulated CD8^+^ splenocytes as compared to splenocytes without iron supplementation indicating that perforin is retained in CD8^+^ cells (p = 0.006) (**Figure 6B**). Moreover, performing chromium release assays, we could demonstrate that CD8^+^ T cells incubated with iron significantly attenuate their ability to kill target cells compared to CD8^+^ T cells without iron application (CD8^+^ T cell:target cell ratio 30:1 p = 0.017) (**Figure 6C**).

The main mechanism of toxicity of chemically reactive cellular iron relies on the excellent redox properties of the element culminating in the generation of reactive oxygen species (1, 42). In line with that, we found a significant accumulation of mitochondrial ROS in CD8^+^ T cells in splenocyte cultures supplemented with TBI or NTBI (CD8^+^ T cells ferric sulfate p <0.05; holo-transferrin p <0.05) as measured by the fluorescent dye MitoSOX. In turn, cytoplasmic ROS formation detected by the DCFDA dye was unaltered by iron stimulation (**Figure 7A**). Interestingly, these effects could not be observed in the CD4^+^ T cells fraction in the same culture (**Figure 7B**). The iron-dependent effect on T cell growth was reversed by the addition of MitoTempo, a mitochondria specific anti-oxidant (iron:cell death/ROS inhibitor interaction CD4^+^ p = 0.009, CD8^+^ p = 0.0026). Other cell death and stress inhibitors like Ferrostatin-1 (inhibits ferroptosis), the cytoplasmic ROS scavenger NAC (cell death/ROS inhibitor), Necrostatin (inhibits necroptosis), or the Casp3i z-DEVD-FMK (inhibits apoptosis; cell death/ROS) showed no significant effects in regard to reversal of iron-mediated impairment of T cell proliferation (**Figures 8A, B**). These results suggest that iron exposition negatively impacts on T cell function by inhibiting CD8^+^ cytotoxic T cells degranulation and perforin-mediated killing of target cells as well as on IFNγ formation by CD4^+^ and CD8^+^ T cells. In addition, iron exposure induces mitochondrial ROS causing growth arrest and cell death of those lymphocytes. This is in line with the reduced efficacy of cancer immunotherapy in iron-administered animals as described herein.

**Figure 9:**
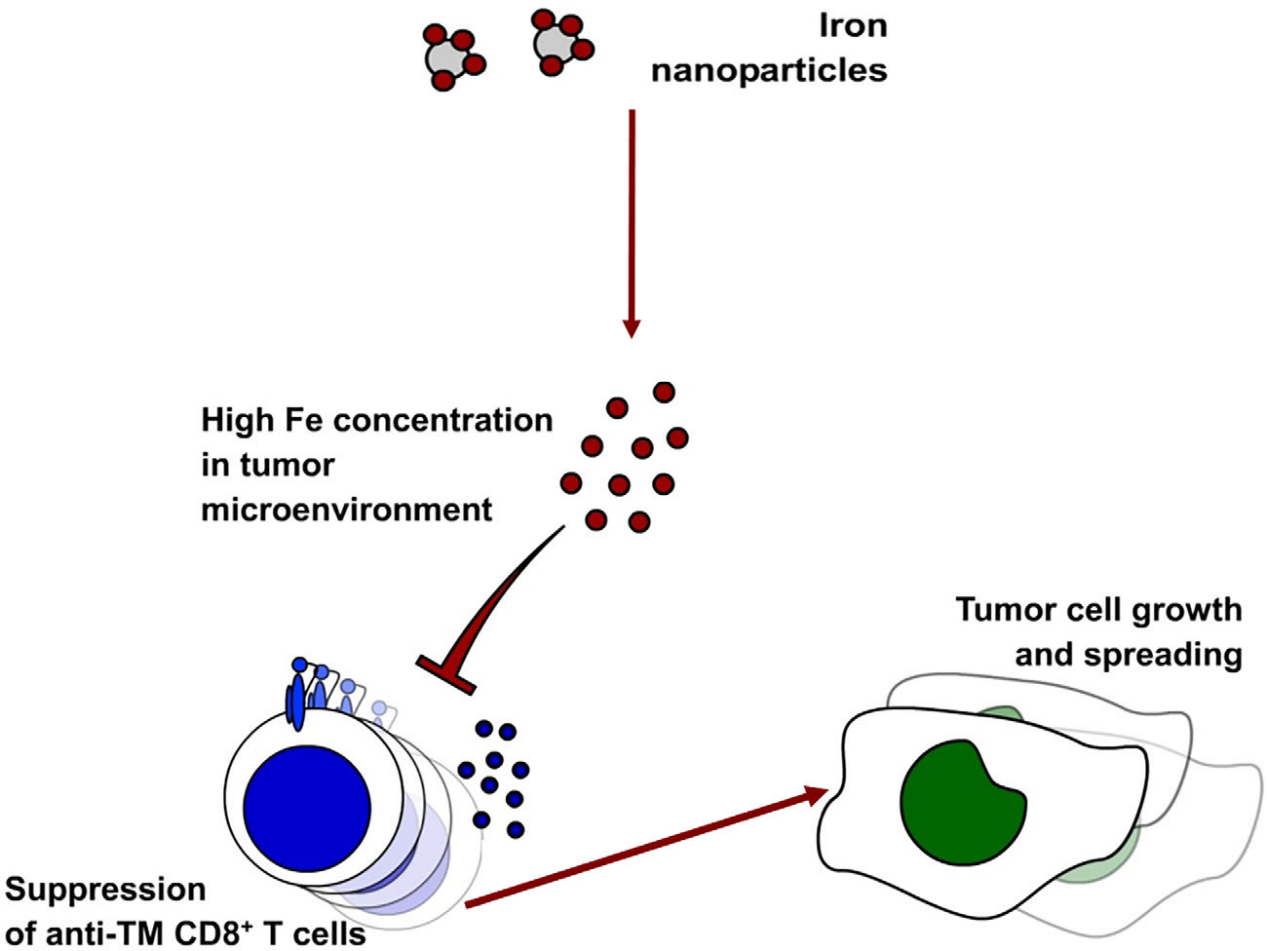
Administration of intravenous iron in the form of ferric isomaltose leads to higher iron concentrations in the tumor milieu. This leads to the inhibition of anti-tumor CD8^+^ T cells.

Taken together, increased iron concentration in the tumor milieu caused by intravenous iron supplementation hampers activation, expansion, survival and functionality of the two key effectors of anti-tumor immunity, CD8^+^ cytotoxic T cells and CD4^+^ T helper cells (**Figure 9**). Our results indicate strong immunosuppressive effects of iron on anti-tumor immunity and on the efficacy of immune-therapies for cancer.

## Discussion

Patients with breast cancer and other malignant diseases often develop functional iron deficiency or overt anemia as a consequence of their underlying disease (10, 11, 48). Anemia per se may negatively affect cardiovascular function and quality of life in affected patients, so that physicians frequently see the necessity to correct anemia by different treatments (34, 35). Intravenous iron administration is one of the treatment options for ACD in oncologic patients specifically if they suffer from absolute iron deficiency with low serum ferritin levels (49) which often coexists based on chronic blood losses and which aggravates the severity of ACD (50, 51). In parallel, intravenous iron preparations improve response rates to and exert dose-sparing effects on the use of erythropoiesis stimulating agents (ESA) (52, 53). This is relevant because treatments with high doses of ESA have been linked to tumor progression as the erythropoietin receptors are expressed on cancer cells including mammary carcinoma, whereas erythropoietin inhibits pro-inflammatory immune responses of innate immune cells which may hamper cancer control (54–57). While the effects of iron treatment on the hematological response have been well studied, no data are available on the effect of such intervention on the clinical course of the underlying tumor disease including end point data (11, 58). Principally, there are several ways by which iron administration may affect the clinical course of breast cancer. First, iron may have direct effects on either the division or the death of breast cancer cells. Consequently, iron can either sustain tumor cell metabolism and promote their proliferation (16, 59) or may sensitize cancer cells to ferroptosis, especially in the context of anti-tumor therapies (60). Second, the administration of iron may impact on the immune control of the tumor and either stimulate or inhibit the activity of distinct immune pathways against malignant cells. Third, iron may affect the susceptibility of tumors cells to immune- or chemotherapy in different ways, either by aggravating radical formation and cancer cell apoptosis/ferroptosis or by inducing their proliferation thereby making them more sensitive to the effects of anti-proliferative agents.

We designed our study to investigate the effects of iron isomaltoside, a clinically approved intravenous iron compound, on the course of disease, therapeutic efficacy of anti-cancer immunotherapies and anti-tumor response of CD4^+^ and CD8^+^ tumor infiltrating T cells in the E0771 breast cancer model (61). Blood counts of untreated tumor-bearing mice point out significantly lower hemoglobin concentrations 21 days after tumor implantation as compared with tumor free-mice, referring to a mild impairment of erythropoiesis, which, partly, recapitulates cancer-associated anemia found in a substancial percent of breast cancer patients.

Our results obtained *in vivo* show that iron isomaltoside accelerates tumor progression in therapy-naive mice as compared with iron-untreated tumor bearers. Of practical relevance for cancer treatment, it also significantly diminished the efficacy of the IL-2/doxorubicin chemo-immunotherapy treatment regimen and substantially, yet not significantly, aggravated the effects of the anti-PD-L1 treatment. Notably, CD8^+^ IFNγ^+^ T cell-mediated anti-tumor response poses one of the mechanisms of action of doxorubicin therapy as demonstrated by us previously (62) and is of critical importance for the anti-PD-L1 immune checkpoint therapy (63, 64). Our results show significant reduction of this T cell population in the tumor tissue of mice treated with either therapy regimen combined with iron, suggesting that inhibition of anti-tumor T cell response poses the common mechanism of the detrimental action of iron supplementation. Another argument for the common mode of action is the cross-talk between the immune checkpoint pathways and signaling induced by IL-2 and doxorubicin. In breast cancer, CD8^+^ T cell numbers correlate with PD-L1 expression (65, 66) because tumor-infiltrating CD8^+^ T cells carry PD-L1 (67). Furthermore, CD8^+^ T cells are required to mediate the anti-tumor effect of PD-L1 blockade against cancer cells, as shown in a mouse model of malignant melanoma (68). In addition, doxorubicin co-administered with cisplatin (the latter not used in our study) upregulates PD-L1 expression in breast cancer (69) and PD-L1 inhibition in combination with IL-2 has synergistic effects on CD8^+^ T cells, suggesting that these two therapies may activate converging pathways (70). Moreover, the PD-L1 and IL-2 pathways are interconnected: On the one hand, the PD-1/PD-L1 interaction inhibits IL-2 production. On the other hand, exogenous IL-2 is known to overcome the inhibitory effects of this interaction (71). Taken together, it is reasonable to assume that iron impairs the anti-tumor effects of anti-PD-L1 antibodies and of IL-2/doxorubicin immunochemotherapy mainly by impairing CD8^+^ T cell functions.

This hypothesis is further supported by the results of *in vitro* experiments clearly demonstrating that increased iron supply, not only as potentially toxic NTBI but also in its physiological transferrin-bound form impairs CD8^+^ T cell proliferation, cytokine production and degranulation. Of interest, the effects of iron isomaltoside on CD4^+^ T cells were less pronounced supporting the fact that the main function of CD4^+^ T cells in the tumor setting is the initiation and maintenance of CD8^+^ tumor infiltrating killer cells or rather to shape the anti-tumor response in spleens and lymph nodes. In line, the administration of iron isomaltoside had consistent yet not significant effects on effector/memory tumor infiltrating lymphocyte populations. In contrast, numbers of FoxP3^+^ CD4^+^ Tregs were comparable across treatment arms suggesting that the adverse effects of iron towards effector T cell populations where direct rather than indirect and Treg-mediated (72, 73).

Our observations raise the question of how iron may impair tumor infiltrating lymphocyte responses in breast cancer-bearing mice. First, iron may impair the proliferation, differentiation or maturation of naïve tumor infiltrating lymphocytes by mitochondrial ROS generation resulting in cell death as indicated by our *in vitro* data. Notably, such a process may take place both in the spleen, which, together with the liver, represent the major storage organ upon ferric isomaltoside treatment, and in the tumor milieu displaying improved iron availability as demonstrated by reduced CD71 levels on the bona-fide neoplastic epithelium. The tendency towards reduced numbers of CD4^+^ effector T cells following iron isomaltoside administration presented in **Figure 2D** may in fact reflect such iron-mediated cell death happening locally in the tumor microenvironment. Second, iron may impair T cell receptor signaling and thus T cell activation. The *in vitro* data on the increased cell death, reduced proliferation and impaired IFNγ production of iron-exposed CD4^+^ and CD8^+^ T cells upon CD3 stimulation support this hypothesis. Third, it is feasible to assume that co-stimulatory pathways are undermined by high iron levels in the tumor microenvironment. Yet, in our *in vitro* system, we did not activate CD28 or other co-stimulatory pathways or study putative effects of iron on down-stream signaling events. Fourth, high iron concentrations in the microenvironment of tumor infiltrating lymphocytes may impair IFNγ output by direct negative effects on the transcription or translation of cytokine genes and mRNAs, respectively (74). This would be in line with the negative effects of iron on IFNγ signaling and IFNγ inducible pathways in macrophages, which impact also on T_H_1/T_H_2 cell differentiation (29, 75, 76). In summary, the administration of iron to mice with mammary carcinoma exacerbated the disease and impaired the therapeutic response to cancer-immunotherapy. Further studies are underway to characterize the molecular mechanisms by which iron administration impacts on anti-tumor T cell responses in our clinically relevant breast cancer model. Nonetheless, iron administration to cancer patients may have multiple adverse effects on the course of the underlying malignant disease. Therefore, prospective trials are needed which investigate those most important questions beyond the correction of hemoglobin levels.

## Data Availability Statement

The raw data supporting the conclusions of this article will be made available by the authors, without undue reservation.

## Ethics Statement

The animal study was reviewed and approved by Federal Ministry Republic of Austria Education, Science and Research.

## Author Contributions

PT participated in the study design, data collection and analysis, and drafted the manuscript. MN, VP, SH, BK, NHK, and VK participated in the data collection and revised the manuscript, NB and IT revised the manuscript. GW and CPO participated in the study design, data collection, analysis, data interpretation, and manuscript preparation. All authors contributed to the article and approved the submitted version.

## Funding

Authors MN and IT are currently receiving a grant from the Austrian Research Fund FWF (P33062, P28302, respectively), author GW received a grant by the Christian Doppler Society and an ERA-NET grant by the FWF (EPICROSS, I-3321), and author CP-O was supported by the Austrian Cancer Society/Tirol (P17006). Authors NB and VP were supported by the doctoral college project W1253 HOROS. For the remaining authors none were declared.

## Conflict of Interest

The authors declare that the research was conducted in the absence of any commercial or financial relationships that could be construed as a potential conflict of interest.

## Abbreviations

ACD, anemia of chronic disease; CFSE, Carboxyfluorescein succinimidyl ester; Ctrl, control; DCFDA, 2′,7′-Dichlorodihydrofluorescein diacetate; DOX, doxorubicin; ESA, erythropoiesis stimulating agents; IL-2, interleukin-2; IFNg, interferon gamma; NAC, N-acetyl cysteine; NTBI, non-transferrin bound iron; PD-L1, programmed death-ligand 1; RBC, red blood cell; ROS, reactive oxygen species; TBI, transferrin bound iron; Tc, cytotoxic T cell; Th, helper T cell; Treg, regulatory T cell.
