# Supplementary material for: Iron Supplementation Interferes With Immune Therapy of Murine Mammary Carcinoma by Inhibiting Anti-Tumor T Cell Function
Source: Front Oncol. 2020 Dec 4;10:584477. doi: 10.3389/fonc.2020.584477 (PMC7746876; doi:10.3389/fonc.2020.584477)
Supplement: Supplementary file 1 [file DataSheet_1.docx]

**Supplementary Figure 1. Hematological status of tumor-free and tumor-bearing animals**

12-week old female C57Bl/6 mice were subcutaneously implanted with E0771 cells (2.5×10^5^ cells per animal, n = 9) or left untreated (n = 9). Complete blood count (CBC) was measured on day 21 after tumor implantation.

Each point in the plot denotes a single animal, bars represent means with SEM. Statistical significance was assessed by two-tailed T test. ns: not significant, *: p < 0.05. RBC: Red blood cell counts, HGB: hemoglobin concentration, HCT: hematocrit. tumor free n=8, tumor bearing n=9.

**Supplementary Figure 2. Systemic and local iron content after intravenous iron supplementation.**

(A, B) 2-week old female C57Bl/6 mice were intravenously injected with iron isomaltoside (2mg elementary iron/animal, n = 4) or left untreated (n = 4). Iron content normalised to protein concentration of the spleen and liver was measured with a colorimetric assay 14 days after iron treatment. (C, D) 12-week old female C57Bl/6 mice were subcutaneously implanted with E0771 cells (2.5×10^5^ cells per animal) and intravenously administered iron isomaltoside (2mg elementary iron/animal, Fe) or PBS on day 3 post implantation. Iron content normalised to protein concentration (C) and surface expression of transferrin receptor 1/CD71 on CD45^-^ bona-fide malignant epithelial cells and CD45^+^ tumor-infiltrating leukocytes (D) were measured 3 weeks post implantation with a colorimetric assay and flow cytometry, respectively. CD71 expression was presented as a percent of CD71-positive cells within the CD45^-^ and CD45^+^ populations and as a change in median fluorescence intensity (ΔMFI) in respect to an isotype staining. Iron measurement: n = 16 controls and n = 17 iron-administered animals, CD71 staining: n = 8 controls and n = 9 iron-administered animals. Each point in the plot denotes a single animal, bars represent means with SEM. Statistical significance was determined by a two-tailed T test (A, B, C, D: percent CD71^+^ within CD45^-^) and the Mann-Whitney U-test (D:  ΔMFI and percent CD71^+^ within CD45^+^).  ns: not significant, *: p < 0.05, **: p < 0.01.
