# Supplementary figures and images for: Iron Supplementation Interferes With Immune Therapy of Murine Mammary Carcinoma by Inhibiting Anti-Tumor T Cell Function
Source: Front Oncol. 2020 Dec 4;10:584477. doi: 10.3389/fonc.2020.584477 (PMC7746876; doi:10.3389/fonc.2020.584477)

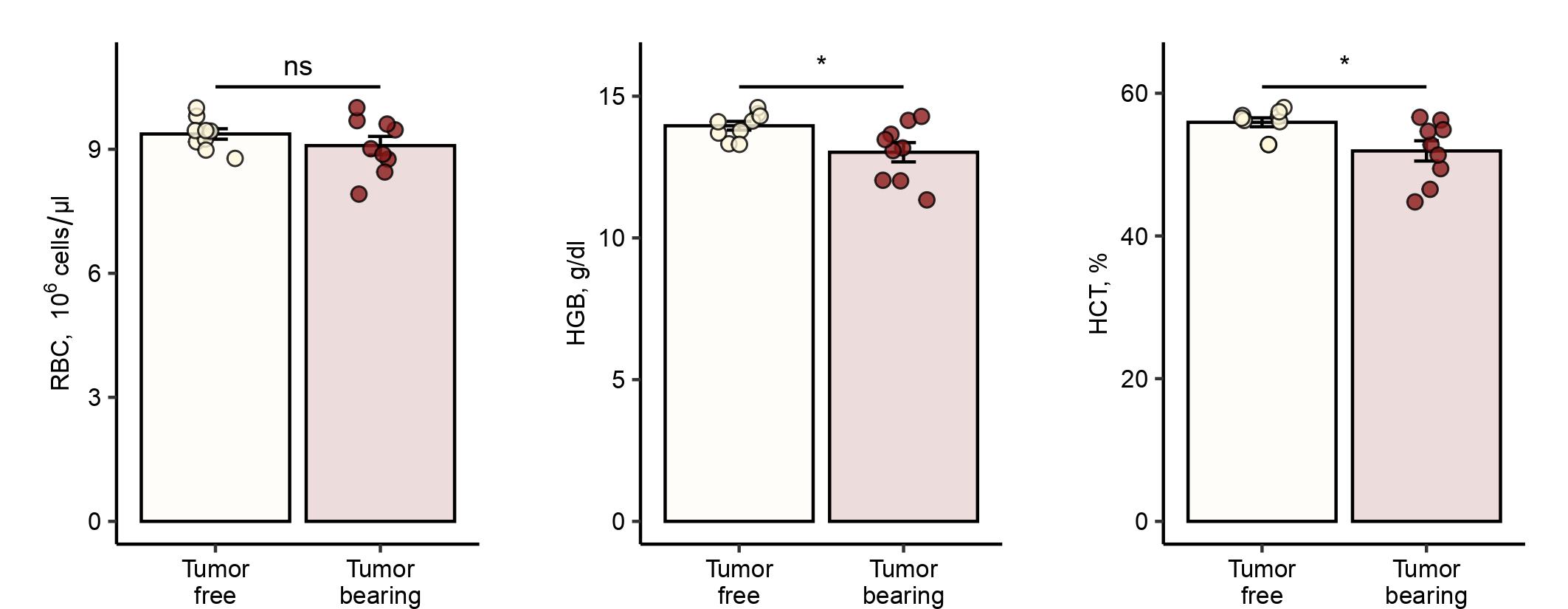

Supplement: Supplementary file 2 [file Image_1.jpeg]

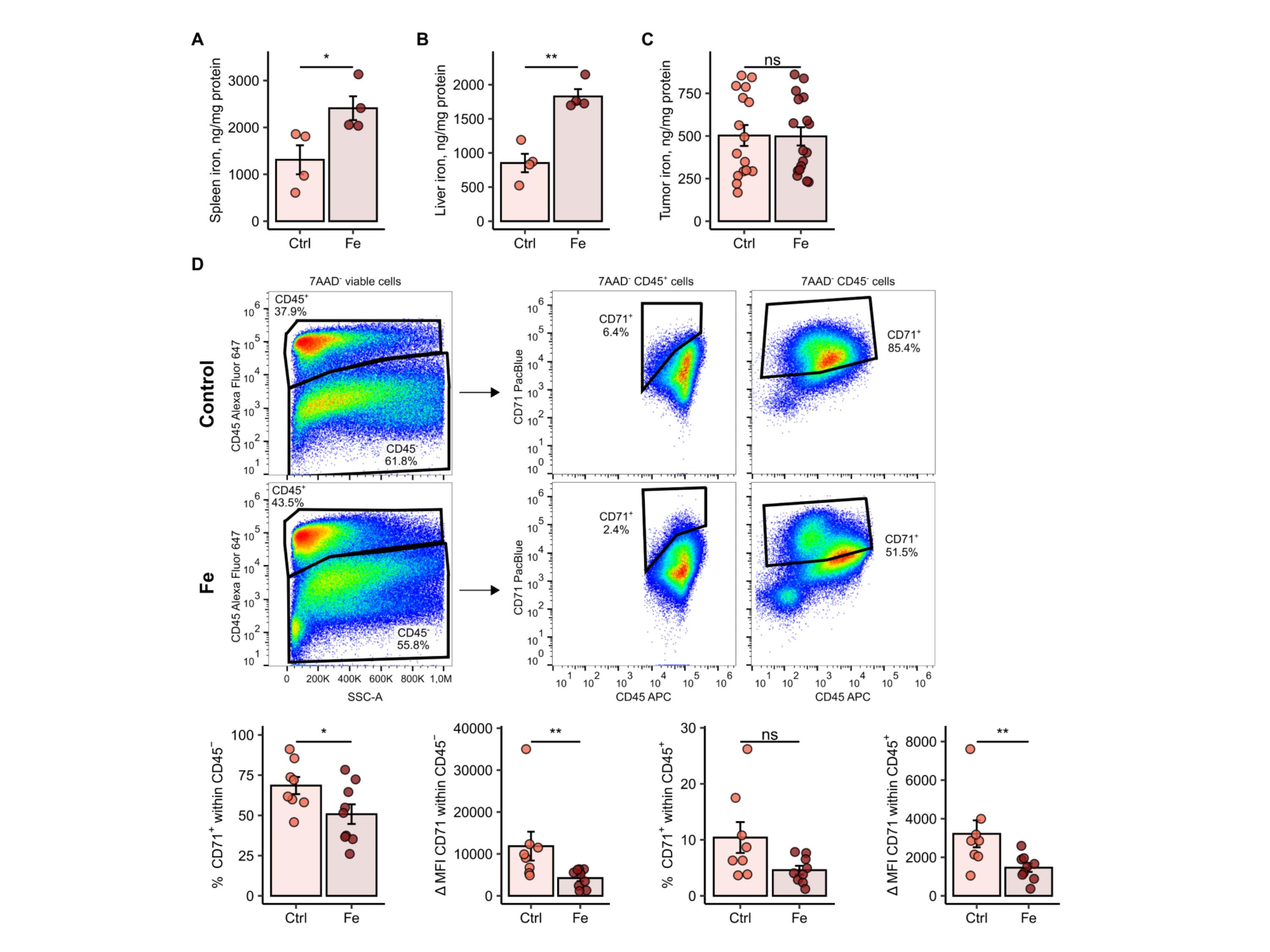

Supplement: Supplementary file 3 [file Image_2.jpeg]
